# Concurrent changes in sleep and cognitive function during retirement transition: the Finnish retirement and aging study

**DOI:** 10.1007/s10433-025-00876-8

**Published:** 2025-08-07

**Authors:** Tea Teräs, Saana Myllyntausta, Jaana Pentti, Jesse Pasanen, Suvi Rovio, Sari Stenholm

**Affiliations:** 1 Department of Public Health, University of Turku and Turku University Hospital, Turku, Finland; 2 Centre for Population Health Research, University of Turku and Turku University Hospital, Turku, Finland; 3 Department of Psychology and Speech-Language Pathology, University of Turku, Turku, Finland; 4 Research Center of Applied and Preventive Cardiovascular Medicine, University of Turku, Turku, Finland; 5 Clinicum, Faculty of Medicine, University of Helsinki, Helsinki, Finland; 6 Research Services, Turku University Hospital and University of Turku, Turku, Finland

**Keywords:** Cognition, Sleep duration, Sleep difficulties, Accelerometry

## Abstract

The transition to retirement has been shown to be accompanied by increased sleep duration and improved sleep quality. In addition, some studies suggest accelerated decline in cognitive function in post-retirement years. However, less is known about their interconnectedness. The aim of this study was to examine the concurrent changes in sleep and cognitive function during retirement transition. The study population consisted of 250 public sector workers (mean age before retirement 63.1 years, standard deviation 1.4) from the Finnish Retirement and Aging study. The participants used a wrist-worn ActiGraph accelerometer, responded to the Jenkins Sleep Problem Scale and underwent cognitive testing annually before and after retirement. Computerized Cambridge Neuropsychological Test Automated Battery (CANTAB®) was used to evaluate learning and memory, working memory, sustained attention and information processing, executive function and cognitive flexibility, and reaction time. Cognitive function improved in all cognitive domains, except for reaction time, during 1-year retirement transition period. The improvement was temporary in learning and memory, working memory and executive function and cognitive flexibility, which plateaued in post-retirement years. The participants were categorized into constantly short (49%), increasing (20%), decreasing (6%), and constantly mid-range (25%) sleep duration; and constantly without (36%), increasing (10%), decreasing (16%), and constantly with (38%) sleep difficulties. There were no associations between changes in sleep duration or sleep difficulties and cognitive function during retirement transition. Cognitive function improves temporarily during transition to retirement, but the improvement is independent of changes in sleep characteristics.

**Supplementary Information:**

The online version contains supplementary material available at 10.1007/s10433-025-00876-8.

## Introduction

Cognitive function changes during one’s lifespan. On average, cognitive function increases in the early years, stabilizes in middle-age, and starts to decline with advancing age (Singh-Manoux et al. 2012; Van Der Willik et al. 2021). The key cognitive changes with normal aging include decline in performance in tasks requiring rapid information processing for decision making, problem solving, and multitasking such as processing speed, working memory, and executive function (Murman 2015). On the other hand, so-called crystalized abilities such as vocabulary, verbal reasoning, and visual recognition are preserved well into old age (Murman 2015). With the aging population, the decrease of cognitive function becomes more apparent, and it would be valuable to find modifiable risk and protective factors affecting cognitive function trajectories with aging.

Retirement is a major life event in late middle-age resulting in numerous changes in everyday life. Retirement has been shown to associate with improved sleep (Hagen et al. 2016; Myllyntausta et al. 2018, 2020) and psychological well-being (Lahdenperä et al. 2022), as well as to modify physical activity behavior (Suorsa et al. 2022). However, the findings regarding changes in cognitive function are inconclusive. Some studies suggest that decline in cognitive function accelerates after retirement with a follow-up ranging from 4 to 18 years (Bonsang et al. 2012; Carr et al. 2020; De Grip et al. 2015; Hamm et al. 2020; Lee et al. 2019; Meng et al. 2017; Oi 2019; Xue et al. 2018). On the other hand, a recent meta-analysis showed that retirement does not associate with global cognitive function, but performance in memory-related tasks slightly decreases after retirement (Alvarez-Bueno et al. 2021). Several factors may explain these mixed findings. First, the length of follow-up as well as the characteristics of the study populations have varied markedly in terms of age and occupational background. Second, previous research has mainly focused on global cognitive function (Lee et al. 2019), a single cognitive domain (Bonsang et al. 2012; Carr et al. 2020), or only a few selected cognitive domains (Hamm et al. 2020; Oi 2019) rather than a wider spectrum of cognitive domains. Moreover, it remains unclear how cognitive function changes during the transition to retirement, as previous research has mainly focused on trends of cognitive function before and after retirement rather than during the actual transition. Consequently, there is a need for further examination to identify subtle changes in different cognitive domains during the retirement transition years among participants from various occupations.

Previous studies have identified several factors that may modify the rate of cognitive decline after retirement including sex (Atalay et al. 2019; Hamm et al. 2020; Oi 2019), occupation (Carr et al. 2020; Finkel et al. 2009; Fisher et al. 2014; Meng et al. 2017), job strain (Nilsen et al. 2021), retirement age (Celidoni et al. 2017; Grotz et al. 2016; Hale et al. 2021), mental activities (Lee et al. 2019), and health (Denier et al. 2017). However, the role of sleep on changing cognitive function during and after retirement is still ambiguous. We have previously shown that increasing and decreasing sleep difficulties are associated with a more pronounced decline in cognitive function during retirement transition during a time span of 5 years (Teräs et al. 2023a, b). Apart from that, although it is known that sleep duration and difficulties associate with cognitive function (Okuda et al. 2021; Swanson et al. 2021; Teräs et al. 2023a, b; Troxel et al. 2022), the role of sleep on changes in cognitive function during the retirement transition has been scarcely examined.

This study aims to expand the previously studied association of retirement on cognitive function by narrowing the follow-up to annual examinations around retirement to study the short-term changes in multiple cognitive domains during the retirement transition. We also aim to examine whether changes in sleep duration and difficulties play a role in the change in cognitive function during retirement transition.

## Methods

### Study population

The study population consisted of the participants of the Finnish Retirement and Aging Study (FIREA), an ongoing longitudinal study of older public sector employees in Finland established in 2013. Detailed description of the FIREA study design and implementation has been reported elsewhere (Stenholm et al. 2023). Shortly, participants were first contacted 18 months prior to their estimated retirement date by sending them a questionnaire. Finnish-speaking participants living in Southwest Finland whose estimated retirement date was between 2017 and 2019, and who were still working were invited to participate in the clinical sub-study (*n* = 773). Of them, 290 participated in the first clinical examination (Teräs et al. 2020). Thereafter, participants were followed up with annual measurements including questionnaires and clinical and accelerometer measurements. To determine the timing of retirement, working status was inquired during an annual clinical visit. Data were then centered around the retirement transition and presented as pre-retirement (waves − 2 to − 1), retirement transition (waves − 1 to 1) and post-retirement (waves 1–2) periods. For this study, the participant had to have information on cognitive function and sleep characteristics from study waves before and after retirement (i.e., waves − 1 and 1). This resulted in a study sample of 250 participants.

Comparison between participants in the clinical sub-study and other FIREA survey participants has been reported previously (Teräs et al. 2020). Briefly, clinical sub-study participants were less likely to have difficulties falling asleep but no other differences in sleep characteristics were observed. There were no differences between self-reported difficulties in memory function. Of the used covariates, the participants in the clinical sub-study were likely to be younger than their counterparts who only took part in survey study, but no other differences were observed.

Informed consent was obtained from all participants. The FIREA study was conducted in accordance with the Helsinki declaration and was approved by the Ethics Committee of Hospital District of Southwest Finland.

### Cognitive function

Cognitive function was evaluated annually during clinical examinations utilizing computerized Cambridge Neuropsychological Test Automated Battery (CANTAB®; https://www.cambridgecognition.com/cantab/). CANTAB is a widely used standardized test battery which comprises of a variety of different cognitive tests covering multiple cognitive domains including working memory; executive function; learning; visual, verbal, and episodic memory; attention, information processing and reaction time; social and emotion recognition, decision making, and response control. The tests used in this study were Paired Associates Learning (PAL) for *visual memory and associative learning* (hereafter *learning and memory*)*,* Spatial Working Memory (SWM) for *working memory*, Rapid Visual Information Processing (RVP) for *sustained attention and information processing*, Attention Switching Task (AST) for *executive function and cognitive flexibility* and Reaction Time (RTI) for *reaction time*. More thorough description of the tests can be found elsewhere (Teräs et al. 2020).

Each CANTAB® subtest produces several outcome variables. For data reduction and to gain summary variables that would explain most of the variation within the data set, we created Z-score based summary scores for each CANTAB subtest. First, each variable was standardized and converted so that a higher value reflects better cognitive function. After that, subtest specific variables were summed and divided by the number of variables from that specific subtest. At baseline, each individual variable was transformed into a scale with a mean of 0 and a standard deviation (SD) of 1. In the follow-up measurements, the standardization was conducted in respect to the baseline distribution (mean and standard deviation) and these values were used to create the summary scores for specific subtests.

### Sleep characteristics

Sleep duration was evaluated objectively with accelerometry. The wrist-worn triaxial wActiSleep-BT accelerometer by ActiGraph (Pensacola, Florida, USA) was initialized to record movements during sleep and wakefulness at 80 Hz. The participants received the device via mail before the cognitive testing and were instructed to wear it continuously on their non-dominant wrist for 24-h per day for at least seven days and nights (including at least two workdays and two free days while still working). The data handling and checking procedures as well as the used algorithms have been described in detail elsewhere (Myllyntausta et al. 2020). Sleep duration was categorized into short (< 7 h), average (7 to < 9 h), and long (≥ 9 h) sleep duration. As there were only two (0.87%) long sleepers before retirement and none after, long-sleepers were excluded from the analyzes regarding sleep duration. Based on participant’s sleep duration immediately before retirement (study wave − 1) and after retirement (study wave + 1), they were then further categorized into (1) constantly short sleep duration, (2) increasing sleep duration (i.e., short sleepers before retirement and average sleepers after), (3) decreasing sleep duration (i.e., average sleepers before retirement and short sleepers after), and (4) constantly average sleep duration.

Sleep difficulties were evaluated with Jenkins Sleep Problem Scale, a four-item survey including questions about difficulties in falling asleep, difficulties in maintaining sleep during the night, waking up too early in the morning, and non-restorative sleep (Jenkins et al. 1988). The response categories for each item were (1) never, (2) 1–3 nights per month, (3) 1 night per week, (4) 2–4 nights per week, (5) 5–6 nights per week, and (6) nearly every night. Items of Jenkins Sleep Problem Scale correspond to the Diagnostic and Statistical Manual of Mental Disorders (DSM) 5 diagnostic criteria for insomnia (excluding non-restorative sleep). (American Psychiatric Association 2013) As the DSM-5 defines insomnia as any of these symptoms occurring at least three nights per week, we similarly defined sleep difficulty as any of the items occurring at least 2–4 night per week. Based on the responses immediately before (study wave − 1) and after retirement (study wave + 1), the participants were then categorized into (1) constantly without sleep difficulties, (2) increasing sleep difficulties (i.e., no difficulties before retirement but difficulties after), (3) decreasing sleep difficulties (i.e., difficulties before retirement but none after), and (4) constantly with sleep difficulties.

### Covariates

The covariates used in this study were age, sex, occupational position, depression, job strain, and alcohol risk use. These covariates were selected based on their known association with sleep characteristics and cognitive function. Covariates were evaluated at the study phase immediately before retirement (study wave − 1).

Participants’ date of birth, sex, and occupational status were obtained from the pension insurance institute for the municipal sector in Finland. Occupational status was categorized based on the International Standard Classification of Occupations (ISCO) into three groups according to the occupational titles: managers and professionals as administrative (ISCO classes 1–2), associate professionals and office workers as professional/executive (ISCO classes 3–4), and service and manual workers as clerical/support (ISCO classes 5–9).

Depression, job strain, and alcohol risk use were obtained from the questionnaires. Depression was evaluated with Beck Depression Inventory (BECK et al. 1961) with a cutoff point of 10 or more indicating mild or severe depression. Job strain was measured using scales of job control and job demands from the shorter version of the Job Content Questionnaire (Fransson et al. 2012; Karasek 1979) using the median values from the entire FIREA cohort as the cutoff points (job control 3.75 and job demands 3.2) to identify the participants with job strain (a high “demands” and a low “control” score). Alcohol risk use was defined as > 16 drinks/week for women and > 24 drinks/week for men corresponding with the lower limit for heavy use of alcohol set by the Finnish Ministry of Health and Social Affairs (Health 2006).

### Statistical analyses

Sample characteristics before retirement are shown as percentages for categorical variables and means and standard deviations (SD) for continuous variables.

To estimate the mean level (95% confidence limits) of each cognitive domain in pre-retirement years (study waves − 2 and − 1) and post-retirement years (study waves + 1 and + 2), we performed linear regression analyses with generalized estimating equations (GEE) with an exchangeable correlation structure. The GEE model controls for intraindividual correlation between repeated measures and assumes that measurements are missing completely at random (Diggle 2002; Zeger and Liang 1986).

Mean levels of each cognitive domain by sleep duration and sleep difficulty status before retirement (wave − 1) were examined with analysis of variance. The association between changes in sleep duration and difficulties and changes in cognitive function during retirement transition (waves − 1 to 1) were examined with GEE models, which included a “sleep group × time (i.e., cognitive function before or after retirement)” interaction term for group comparisons. The analyses were initially adjusted for age, sex, and occupational position. The analyses were further adjusted for depression, job strain, and alcohol risk use.

All statistical analyses were performed using SAS version 9.4 (SAS Institute Inc., Cary, NC, USA).

## Results

The pre-retirement (study wave − 1) characteristics of the study population are shown in Table 1. The mean age of the participants at the study phase before retirement was 63.1 years (SD 1.1), and the majority of participants were woman (84%). Each occupational group was represented rather equally: 35% of the participants were in administrative, 34% in professional/executive, and 32% in the clerical/support occupations. Impaired sleep was relatively common before retirement: 53% of the participants reported sleep difficulties, and 68% were short sleepers.

**Table 1:** Characteristics of the study population before retirement (*N* = 250)

	Mean	SD
Age, years	63.12	1.09

	n	%
Sex		
Male	40	16
Female	210	84
Occupational position		
Administrative	87	35
Professional/executive	84	34
Clerical/support	79	32
Depression	28	11
Job strain	43	19
Alcohol risk use	25	10
Sleep difficulties	131	53
Sleep duration		
< 7 h	162	68
7 to < 9 h	74	31
≥ 9 h	2	0.8

**Fig. 1:**
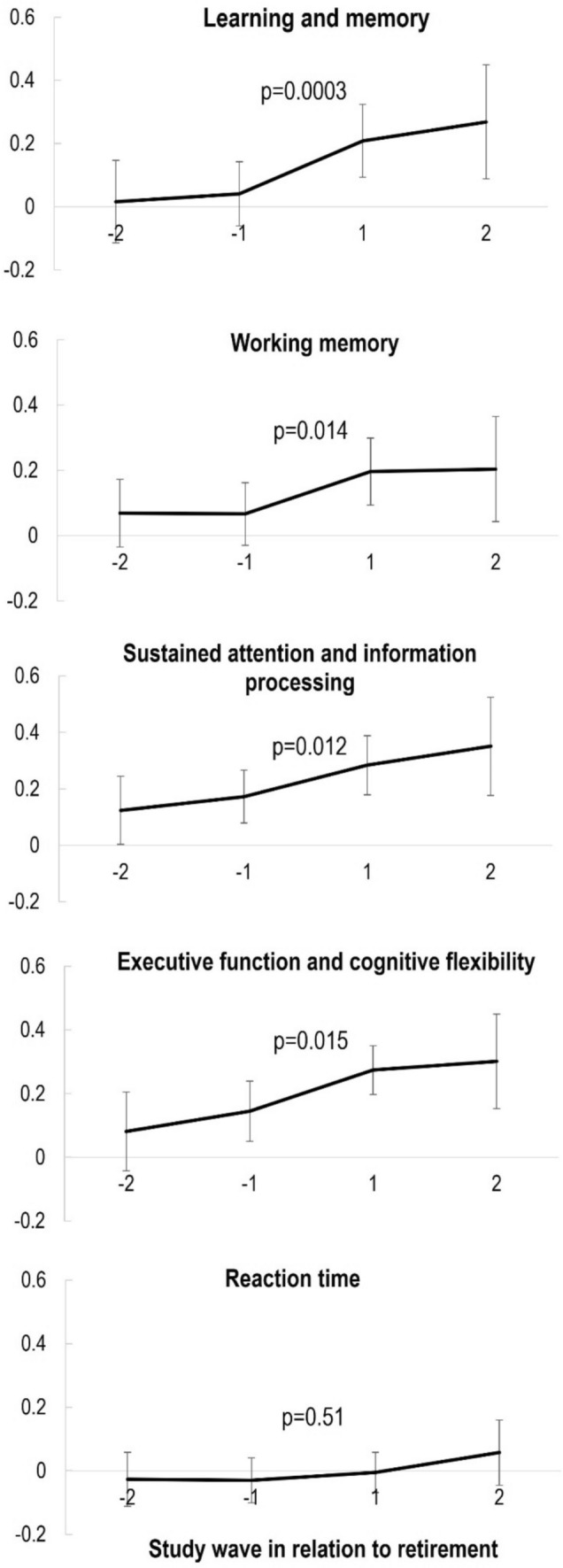
Mean level and their 95% confidence intervals in each cognitive domain before and after retirement. Adjusted for age, sex, and occupational position. The p-values that are shown describe the change in cognitive function during the retirement transition (wave − 1 to wave + 1)

The changes in each cognitive domain before, during, and after retirement transition are shown in Fig. 1. In terms of *learning and memory*, there was no change in pre-retirement (mean change in pre-retirement 0.025, *p* = 0.64), but a clear improvement during the retirement transition (mean change in retirement transition 0.17, *p* = 0.0030), which plateaued after retirement. The trends in pre-retirement and retirement transition differed statistically significantly (*p* = 0.026). Similarly, *working memory* remained stable in pre-retirement (mean change in pre-retirement − 0.003, *p* = 0.96), but improved during the retirement transition (mean change in retirement transition 0.13, *p* = 0.014), and remained stable during first post-retirement years. However, the difference in the trends in pre-retirement and retirement transition did not quite reach statistical significance (*p* = 0.091). *Sustained attention and information processing* as well as *executive function and cognitive flexibility* improved throughout the follow-up retirement transition (mean change in pre-retirement 0.048, *p* = 0.29; mean change in the retirement transition 0.11, *p* = 0.012 and mean change in pre-retirement 0.064, *p* = 0.13; mean change in the retirement transition 0.13, *p* = 0.015, respectively). The difference in trends was nonsignificant (*p* = 0.47 for *sustained attention and information processing*; and *p* = 0.66 for *executive function and cognitive flexibility*) *Reaction time* stayed at the same level before and after retirement and no improvement was observed during retirement transition (mean change in pre-retirement − 0.004, *p* = 0.93; mean change in the retirement transition 0.02, *p* = 0.51). When further adjusted for depression, job strain, and alcohol consumption the results remained similar. Supplemental Table 1 shows mean changes of cognitive function during the retirement transition by occupational status and sex. There were no differences between occupational status groups in the change of cognitive function. Some sex differences were observed, although the results must be interpreted with caution due to small number of men (*n* = 40). Among women, learning and memory improved more than among men (*p* for interaction 0.0066), and among men, reaction time improved more than among women (p for interaction 0.040). Table 2 shows the pre-retirement association of sleep and cognitive domains. Those with mid-range sleep had better performance in *executive function and cognitive flexibility* than short sleepers (*p* = 0.014). There was also a tendency toward better *working memory* and *sustained attention and information processing* among mid-range sleepers compared to short sleepers, but these differences did not reach statistical significance level 0.05. On the contrary, those with short sleep duration had better *learning and memory* than mid-range sleepers (*p* = 0.044). There were no differences in the level of cognitive domains between those with and without sleep difficulties before retirement.

**Table 2:** Mean levels of each cognitive domain by sleep duration and sleep difficulty status before retirement

	Mean	95% CI		*p* value
*Learning and memory*				
Short sleep duration	0.11	− 0.02	0.23	0.044
Mid-range sleep duration	− 0.08	− 0.26	0.09	
No sleep difficulties	0.08	− 0.06	0.21	0.469
Sleep difficulties	0.02	− 0.13	0.16	
*Working memory*				
Short sleep duration	0.07	− 0.05	0.18	0.092
Mid-range sleep duration	0.21	0.06	0.36	
No sleep difficulties	0.13	0.01	0.25	0.504
Sleep difficulties	0.07	− 0.07	0.21	
*Sustained attention and information processing*				
Short sleep duration	0.15	0.04	0.26	0.059
Mid-range sleep duration	0.31	0.16	0.47	
No sleep difficulties	0.18	0.05	0.31	0.812
Sleep difficulties	0.20	0.08	0.31	
*Executive function and cognitive flexibility*				
Short sleep duration	0.11	0.01	0.21	0.014
Mid-range sleep duration	0.30	0.19	0.41	
No sleep difficulties	0.15	0.03	0.27	0.966
Sleep difficulties	0.15	0.03	0.28	
*Reaction time*				
Short sleep duration	− 0.06	− 0.17	0.04	0.512
Mid-range sleep duration	− 0.02	− 0.14	0.10	
No sleep difficulties	− 0.07	− 0.18	0.03	0.527
Sleep difficulties	− 0.03	− 0.15	0.08	

Adjusted for age, sex, and occupational position. Group sizes are: short sleep duration *n* = 157, mid-range sleep duration *n* = 71, no sleep difficulties *n* = 115, and sleep difficulties *n* = 127

In terms of changes in sleep during retirement transition, 49% had constantly short sleep duration, 19% increased, 7% decreased, and 25% had constantly mid-range sleep duration. In addition, 36% remained without sleep difficulties, 10% increased, 16% decreased, and 38% had constantly sleep difficulties. Age, sex, and occupational position adjusted results for changes in the studied cognitive domains during retirement transition within the sleep duration change groups are shown in Table 3. No statistically significant differences between the sleep duration change groups were observed. The results remained similar when further adjusted for depression, job strain and alcohol consumption. Similarly, Table 4 shows the age, sex, and occupational position adjusted results for changes in the cognitive domains during retirement transition in the sleep difficulty change groups. Again, no statistically significant differences were found between the groups, and the further adjustments for depression, job strain, and alcohol risk use did not change the results.

**Table 3:** Mean change in each cognitive domain during retirement transition by sleep duration change groups

	Retirement transition			
	Mean change	95% CI		*p* for interaction
*Learning and memory*				0.483
Constantly short sleep	0.15	0.03	0.26	
Increasing sleep duration	0.16	0.02	0.30	
Decreasing sleep duration	0.27	0.10	0.45	
Constantly mid-range sleep	0.24	0.08	0.39	
*Working memory*				0.628
Constantly short sleep	0.16	0.00	0.31	
Increasing sleep duration	0.23	0.03	0.44	
Decreasing sleep duration	0.16	− 0.18	0.49	
Constantly mid-range sleep	0.09	− 0.04	0.22	
*Sustained attention and information processing*				0.375
Constantly short sleep	0.11	0.00	0.22	
Increasing sleep duration	0.16	0.04	0.27	
Decreasing sleep duration	0.20	0.00	0.39	
Constantly mid-range sleep	0.03	− 0.10	0.16	
*Executive function and cognitive flexibility*				0.329
Constantly short sleep	0.13	0.02	0.24	
Increasing sleep duration	0.23	− 0.03	0.48	
Decreasing sleep duration	0.05	− 0.05	0.14	
Constantly mid-range sleep	0.11	0.00	0.22	
*Reaction time*				0.735
Constantly short sleep	0.05	− 0.06	0.17	
Increasing sleep duration	− 0.05	− 0.17	0.08	
Decreasing sleep duration	0.00	− 0.25	0.25	
Constantly mid-range sleep	0.02	− 0.11	0.15	

Adjusted for age, sex, and occupational position. Group sizes are: constantly short sleep *n* = 111, increasing sleep duration *n* = 44, decreasing sleep duration *n* = 15, and constantly mid-range sleep *n* = 58

**Table 4:** Mean change in each cognitive domain during retirement transition by sleep difficulty change groups

	Retirement transition			
	Mean change	95% CI		*p* for interaction
*Learning and memory*				0.574
Constantly without sleep difficulties	0.15	0.02	0.27	
Increasing sleep difficulties	0.06	− 0.13	0.25	
Decreasing sleep difficulties	0.18	− 0.01	0.36	
Constantly with sleep difficulties	0.21	0.09	0.32	
*Working memory*				0.420
Constantly without sleep difficulties	0.14	− 0.01	0.29	
Increasing sleep difficulties	0.10	− 0.05	0.26	
Decreasing sleep difficulties	− 0.01	− 0.23	0.21	
Constantly with sleep difficulties	0.19	0.05	0.32	
*Sustained attention and information processing*				0.346
Constantly without sleep difficulties	0.17	0.04	0.29	
Increasing sleep difficulties	0.16	− 0.06	0.38	
Decreasing sleep difficulties	0.07	− 0.07	0.22	
Constantly with sleep difficulties	0.05	− 0.06	0.16	
*Executive function and cognitive flexibility*				0.523
Constantly without sleep difficulties	0.14	0.01	0.27	
Increasing sleep difficulties	0.03	− 0.12	0.18	
Decreasing sleep difficulties	0.24	− 0.04	0.52	
Constantly with sleep difficulties	0.12	0.02	0.21	
*Reaction time*				0.299
Constantly without sleep difficulties	0.08	− 0.03	0.19	
Increasing sleep difficulties	− 0.02	− 0.16	0.11	
Decreasing sleep difficulties	0.11	− 0.04	0.26	
Constantly with sleep difficulties	− 0.03	− 0.16	0.09	

Adjusted for age, sex, and occupational position. Group sizes are: constantly without sleep difficulties *n* = 88, increasing sleep difficulties *n* = 23, decreasing sleep difficulties *n* = 39, and constantly sleep difficulties *n* = 92

## Discussion

In this study of retiring workers, we studied how cognitive function changes during retirement transition by addressing various cognitive domains. Additionally, we studied whether changes in sleep duration and difficulties are associated with the changes in different cognitive function domains. By utilizing annually collected data on cognitive function, we showed that cognitive function improves during the transition to retirement. Of the studied cognitive domains, this transient improvement was evident in learning and memory as well as in working memory. In addition, sustained attention and information processing as well as executive function and cognitive flexibility showed improvement throughout the follow-up from pre-retirement years to first post-retirement years. No change was observed in reaction time. When studying the role of sleep in the changes in cognitive function, we found that changes in sleep duration or sleep difficulties were not associated with the observed improvements in cognitive function.

The improved cognitive function during retirement transition is an interesting finding, as it would suggest that something hinders the employees from reaching their full cognitive potential while still in employment. The finding is in line with one previous study, (Celidoni et al. 2017) although most previous studies have found no such effect. The discrepancy in our and previous findings could be due to the different time frame of cognitive measurements as in most studies measurements have been biennial or even with longer follow-ups as their focus has been more on post-retirement years (Atalay et al. 2019; Carr et al. 2020; De Grip et al. 2015; Denier et al. 2017; Finkel et al. 2009; Fisher et al. 2014; Grotz et al. 2016; Hale et al. 2021; Hamm et al. 2020; Lee et al. 2019; Nilsen et al. 2021; Oi 2019; Xue et al. 2018). Celidoni et al. showed in a multi-national European study sample of over 50-year-olds that statutory retirement is at first beneficial to cognitive function, but it starts to decline in post-retirement years (Celidoni et al. 2017). Similarly, Bonsang et al. showed that the negative effect of retirement on cognitive function is not instantaneous in a study sample of over 50-year-olds living in the USA, but found no improvement during retirement transition (Bonsang et al. 2012). Our results are in line with Celidoni et al. and Bonsang et al. but, they both focused only on memory function. The added value of our study is that we were able to study changes in multiple cognitive function domains including learning and memory, working memory, sustained attention and information processing, as well as executive function and cognitive flexibility, and reaction time, and found that performance in all studied cognitive domains, apart from reaction time, improved during retirement transition.

In the current study, we also examined whether sleep duration or sleep difficulties would be associated with short-term changes in cognitive function during retirement transition, since sleep duration has shown to increase and sleep difficulties to decrease during retirement transition (Myllyntausta et al. 2020; Roberts et al. 2011; Vahtera et al. 2009; Xue et al. 2018). Moreover, only few previous studies have linked sleep duration (Teräs et al. 2023a, b; Troxel et al. 2022; Zhang et al. 2021) and sleep difficulties (Teräs et al. 2020; Troxel et al. 2022) to changes in cognitive function. However, we did not find any differences between the sleep duration or difficulty groups in terms of changes in cognitive function. This was somewhat unexpected, as we have previously shown, by using the long-term Whitehall II study, that increasing and decreasing sleep difficulties are associated with accelerated decline in cognitive function during retirement transition (Teräs et al. 2023a, b). However, in that study the cognitive function measurements were 5 years apart and we observed cognitive function to decline only after post-retirement retirement years. Additionally, cognitive function was only evaluated in relation to inductive reasoning and verbal memory rather than a wider range of cognitive domains as was done in this study. The findings of the current study suggest that other factors than sleep characteristics explain the observed change in cognitive function during retirement transition, and this warrants further examination.

This study has several strengths. With annual examinations, we were able to focus more specifically on the transition to retirement unlike most previous studies. Cognitive function was measured with computerized standardized test battery, which can detect even small changes in cognitive function, and allowed us to study multiple individual cognitive domains. Additionally, we were able to utilize objective information on accelerometer-measured sleep duration, which provides more accurate estimation of one’s sleep duration.

There are also some limitations that need to be addressed. As the cognitive function measurements were repeated annually. One previous study has suggested that in CANTAB tests *Learning and memory* and *Working memory* might be subjects to practice effect on frequently repeated measures (Cacciamani et al. 2018). However, practice effect would not explain the improvement seen during the retirement transition in this study, because improvement was observed only in retirement transition but not in pre-retirement years. Additionally, it has been shown that the test–retest reliability within CANTAB is similar to other cognitive tests (Lowe and Rabbitt 1998; Skirrow et al. 2022). Furthermore, compared to the traditional non-computerized test batteries, computerized batteries are also more precise in recording, for example, latency times and accuracy. Simultaneously, they can be more easily optimized so that factors such as ceiling effect does not violate the data, and thus, computerized tests are specifically suitable for relatively cognitively healthy cohorts like ours. There are some additional unmeasured confounding factors such as educational level, which have been shown to be associated with cognitive function (Kremen et al. 2019), but that may have been overlooked in our study. However, the analyses were adjusted for pre-retirement occupational status, which well represents socioeconomic status in this age group. Finally, given that the FIREA clinical study population was healthier compared to the FIREA survey-only participants (Stenholm et al. 2023) and they were able to work until moving to statutory retirement, the generalizability of the findings to aging workers in general may be limited. Additionally, we observed some differences in sleep characteristics and cognitive function between men and women, but the small proportion of men in the sample challenges the interpretation of these results as well as the generalizability of the results overall. Further research with larger sample sizes, more balanced sex distribution, and longer follow-up is warranted to elucidate changes in cognitive function during retirement transition and its determinants.

## Conclusions

We found cognitive function to improve during the retirement transition. This improvement was independent from changes in sleep duration and sleep difficulties. This finding suggests that employees have unused cognitive reserve, and future studies should focus on determinants behind the now observed changes in cognitive function. Identifying targetable determinants behind this change might allow employees to capitalize the unused cognitive reserve when still in working life and therefore improve their productivity at work or even prolong their work-lives.

## Supplementary Information

Below is the link to the electronic supplementary material.

Supplementary file1 (DOCX 24 KB)

## Data Availability

Anonymized partial datasets of the FIREA study are available by application with bona fide researchers with an established scientific record and bona fide organizations. In case of data requests, please contact the principal investigator Sari Stenholm, sari.stenholm@utu.fi.
