# Supplementary material for: Concurrent changes in sleep and cognitive function during retirement transition: the Finnish retirement and aging study
Source: Eur J Ageing. 2025 Aug 7;22(1):40. doi: 10.1007/s10433-025-00876-8 (PMC12331549; doi:10.1007/s10433-025-00876-8)
Supplement: Supplementary file 1 — Supplementary file1 (DOCX 24 KB) [file 10433_2025_876_MOESM1_ESM.docx]

**Supplemental material**

**Concurrent changes in sleep and cognitive function during retirement transition: the Finnish Retirement and Aging Study**

Tea Teräs, MD^1,2^, Saana Myllyntausta, PhD^3^, Jaana Pentti, BSc^1,2,5^, Jesse Pasanen, MSc^1,2^, Suvi Rovio, PhD^1,2,4^, Sari Stenholm, PhD^1,2,6^

^1^ Department of Public Health, University of Turku and Turku University Hospital, Turku, Finland;

^2^ Centre for Population Health Research, University of Turku and Turku University Hospital; Turku, Finland;

^3^ Department of Psychology and Speech-Language Pathology, University of Turku, Turku, Finland;

^4^ Research Center of Applied and Preventive Cardiovascular Medicine, University of Turku, Turku, Finland;

^5^ Clinicum, Faculty of Medicine, University of Helsinki, Finland

^6^ Research Services, Turku University Hospital and University of Turku, Finland

Corresponding author:

Tea Teräs, MD

Department of Public Health, University of Turku, Turku, Finland

Email: [tea.t.teras@utu.fi](mailto:tea.t.teras@utu.fi)

**Supplemental Table 1** Mean change in each cognitive domain during retirement transition by occupational status groups and sex

|  | Retirement transition | | |  |
| --- | --- | --- | --- | --- |
|  | Mean change | 95% CI | | p for interaction |
| **Learning and memory** |  |  |  |  |
| Occupational position |  |  |  | 0.377 |
| Administrative | 0.13 | 0.00 | 0.26 |  |
| Professional / executive | 0.15 | 0.02 | 0.27 |  |
| Clerical / support | 0.23 | 0.11 | 0.35 |  |
| Sex |  |  |  | 0.007 |
| Men | -0.03 | -0.19 | 0.13 |  |
| Women | 0.20 | 0.11 | 0.30 |  |
|  |  |  |  |  |
| **Working memory** |  |  |  |  |
| Occupational position |  |  |  | 0.994 |
| Administrative | 0.13 | -0.01 | 0.27 |  |
| Professional / executive | 0.13 | 0.00 | 0.27 |  |
| Clerical / support | 0.12 | -0.06 | 0.30 |  |
| Sex |  |  |  | 0.114 |
| Men | 0.01 | -0.15 | 0.16 |  |
| Women | 0.15 | 0.04 | 0.26 |  |
|  |  |  |  |  |
| **Sustained attention and information processing** |  |  |  |  |
| Occupational position |  |  |  | 0.644 |
| Administrative | 0.09 | -0.02 | 0.20 |  |
| Professional / executive | 0.10 | -0.02 | 0.23 |  |
| Clerical / support | 0.15 | 0.02 | 0.28 |  |
| Sex |  |  |  | 0.300 |
| Men | 0.06 | -0.05 | 0.16 |  |
| Women | 0.12 | 0.03 | 0.22 |  |
|  |  |  |  |  |
| **Executive function and cognitive flexibility** |  |  |  |  |
| Occupational position |  |  |  | 0.984 |
| Administrative | 0.12 | 0.02 | 0.23 |  |
| Professional / executive | 0.13 | 0.01 | 0.25 |  |
| Clerical / support | 0.14 | -0.05 | 0.32 |  |
| Sex |  |  |  | 0.121 |
| Men | 0.06 | -0.04 | 0.15 |  |
| Women | 0.14 | 0.03 | 0.26 |  |
|  |  |  |  |  |
| **Reaction time** |  |  |  |  |
| Occupational position |  |  |  | 0.243 |
| Administrative | 0.10 | -0.02 | 0.21 |  |
| Professional / executive | -0.01 | -0.13 | 0.12 |  |
| Clerical / support | -0.03 | -0.13 | 0.08 |  |
| Sex |  |  |  | 0.040 |
| Men | 0.21 | 0.02 | 0.40 |  |
| Women | -0.01 | -0.09 | 0.07 |  |

Adjusted for age, sex (analyses regarding occupational position), and occupational position (analyses regarding sex). Group sizes in occupational position: Administrative 87, Professional / executive 84, Clerical / support 79. Group sizes in sex: men 40, women 210.
